# Inhibition of YAP suppresses CML cell proliferation and enhances efficacy of imatinib in vitro and in vivo

**DOI:** 10.1186/s13046-016-0414-z

**Published:** 2016-09-06

**Authors:** Hui Li, Zhenglan Huang, Miao Gao, Ningshu Huang, Zhenhong Luo, Huawei Shen, Xin Wang, Teng Wang, Jing Hu, Wenli Feng

**Affiliations:** 1 Department of Clinical Hematology, Key Laboratory of Laboratory Medical Diagnostics Designated by the Ministry of Education, Chongqing Medical University, No.1, Yixueyuan Road, Chongqing, 400016 China; 2 Department of Hematology, The First Affiliated Hospital, Chongqing Medical University, Chongqing, 400016 China

**Keywords:** Chronic myeloid leukemia, Bcr/Abl, YAP, Verteporfin, IM, Leukemogenesis

## Abstract

**Background:**

Yes-associated protein (YAP), an essential component of Hippo pathway, was identified as an oncoprotein which participated in the progression of various malignancies. However, its role in chronic myeloid leukemia (CML) remains to be further clarified.

**Methods:**

The expression of YAP in CML cells was determined by western blotting. Next, the effects of YAP knockdown and YAP inhibitor on CML cells were evaluated by MTT assay, flow cytometry (FCM) and Wright’s staining. Moreover, K562 induced mice model was employed to further investigate the role of YAP in vivo.

**Results:**

YAP was overexpressed in CML cells. Knockdown of YAP by si-RNA or inhibition the function of YAP using verteporfin (VP) not only inhibited the proliferation, induced the apoptosis of CML cells but also reduced the expression of YAP target genes c-myc and survivin. Additionally, VP enhanced the efficacy of imatinib (IM) in vitro and suppressed leukemogenesis in vivo.

**Conclusion:**

Our results indicate that YAP may play an important role in the proliferation and leukemogenesis of CML cells. Genetic or pharmacological inhibition of YAP provides a novel treatment strategy for CML.

**Electronic supplementary material:**

The online version of this article (doi:10.1186/s13046-016-0414-z) contains supplementary material, which is available to authorized users.

## Background

Chronic myeloid leukemia (CML) is a clonal disorder characterized by BCR/ABL, a constitutively activated tyrosine kinase generated from the reciprocal translocation between chromosomes 9 and 22 [1–5]. BCR/ABL activates multiple pathways involved in the regulation of cell proliferation and apoptosis such as PI3K-AKT [6], MEK-ERK [7, 8] and STAT5 [9], resulting in progressive granulocytosis. Patients newly diagnosed with CML are commonly treated with inhibitor of BCR/ABL named imatinib (IM) [10]. However, tyrosine kinase inhibitors (TKIs)-resistance due to the occurrence of mutations and the limited action of TKIs in patients with blast crisis have emerged as significant clinical issues [11]. Therefore, it is urgent to find more efficient therapeutic strategies to overcome these problems.

Hippo pathway, firstly discovered in Drosophila, is evolutionarily conserved in mammals. This pathway plays an important role in controlling organ size, regulating self-renewal and differentiation of stem cells [12–14]. In vertebrates, the Hippo pathway functions through a kinase cascade comprises of two kinases Mst and Lats, and their co-factors WW45 and Mob. Normally, when cells proliferate to a high density, Hippo pathway is activated [15]. First, Mst1/2 forms a complex with Sav1, and then the complex phosphorylates Lats1/2. Activated Lats1/2 further phosphorylates YAP on Ser127 and promotes its cytoplasmic retention and subsequent degradation by ubiquitin-proteasome pathway [16, 17].

Unfortunately, Hippo pathway has been found to be inactive in various kinds of malignant tumors [18–20]. In these tumor cells, YAP can not be phosphorylated and degraded effectively. Unphosphorylated YAP enters into the nucleus where YAP binds and activates transcription factors, altering the expression of genes involved in cell proliferation and apoptosis [21–23]. In addition, YAP has been identified as an oncoprotein elevated in cholangiocarcinoma [24], ovarian cancer [25], colorectal cancer [26], hepatocellular carcinoma [27] and gastric cancer [28]. YAP has also been found to act as a stem cell regulator [29, 30] and is highly expressed in the stem cell fractions [31]. Moreover, it has been revealed that the expression of YAP was significantly higher in CLL (chronic lymphoblastic leukemia) patients than that in health donors [32]. Although it has been demonstrated that YAP – induced apoptosis was mediated by the aberrant presence of ABL1 in the nucleus in MM cells [33], in CML cells where ABL1 was commonly translocated, the function of YAP was unclear. Considering that c-Myc and survivin, target genes of both BCR/ABL and Hippo-YAP pathways which are associated with the regulation of cell proliferation, are involved in the progression and response to IM in CML [34–37], the role of YAP in the pathogenesis of CML may be interesting to explore.

In this study we found that YAP was highly expressed in bone marrow mononuclear cells (BMMNCs) from CML patients and CML cell lines. We also found knockdown of YAP inhibited the proliferation and induced apoptosis of CML cells. Importantly, we demonstrated that inhibition of YAP by veterporfin (VP) significantly increased the efficacy of IM in vitro and in vivo. Taken together, this is the first report which examined the role of YAP in CML and the effect of YAP inhibition on the response of CML cells to IM. It may provide a feasible therapeutic strategy in the treatment of CML.

## Methods

### Cell culture

The 32DP and BP210 cell lines were generated from 32D and BaF3 cell lines respectively by stably transformed by p210^BCR-ABL^. K562, K562/G01, 32DP, BP210, HL-60, THP1 and NB4 cells were maintained in RPMI-1640 (Gibco, USA) supplemented with 10 % fetal bovine serum (Gibco, USA). For 32D and Ba/F3 cells, 1 ng/ml of murine IL-3 (PeproTech, USA) was added to the medium. All cells were cultured at 37 °C in a humidified atmosphere with 5 % CO_2_.

### Clinical samples

Bone marrow (BM) of normal individuals (5 cases) and CML patients (9 cases) were obtained from the first affiliated hospital of Chongqing Medical University, Chongqing, China. Mononuclear cells were isolated using human bone marrow mononuclear cells isolation kit (Tbd science, Tianjin, China).

### Small molecules, siRNA, and antibodies

The siRNA targeting YAP and the non-targeting siRNA were purchased from Ruibobio (Guangzhou, China). The target sequences for YAP siRNA are 5′-GCGUAGCCAGUUACCAACA dTdT-3′, 5′- CAGUGGCACCUAUCACUCU dTdT-3′ and 5′- GGUGAUACUAUCAACCAAA dTdT-3′. IM was obtained from Novartis (Basel, Switzerland) and VP from Selleckchem (Houston, TX). Following antibodies were used in this study: anti-YAP(S127), anti-YAP(S397), anti-Bax, anti-caspase-3, anti-PARP, anti-p21 (Cell Signaling Technology, USA); anti-YAP and the HRP-conjugated secondary antibodies (Santa Cruz Biotechnology, USA); anti-CyclinD1, anti-survivin and anti-c-Myc (Bioworld Technology Inc, USA); anti-β-Actin (Zhong shan jin qiao, China).

### Small molecule treatment and RNA interference

2 × 10^5^ cells were plated in 6-well plates and transfected with 50 pmol of siRNA using Lipofectamine 2000 (Invitrogen, NY, USA) according to the manufacturer’s protocol. After transfection for 48 h, cells were harvested for viability, cell cycle and apoptosis analysis. Small molecule inhibitors IM and VP were dissolved in DMSO. Cells treated with these inhibitors were collected at indicated time for further analysis.

### Western blotting

Cells were collected and lysed by RAPI lysis buffer supplemented with proteinase and phosphatase inhibitors (Cell Signal, USA) at 4 °C for 20 min. After centrifuged at 4 °C for 15 min at 13,000 g the supernatants were collected. Then equal amounts of extracts (60 μg) were separated by 8–10 % SDS-PAGE and transferred onto the PVDF membranes (Millipore, Boston, MA, USA), and was blocked in 5 % nonfat milk/TBST, incubated with indicated antibodies overnight at 4 °C, followed by incubation with HRP-conjugated secondary antibody for 1 h at 37 °C. Detection was performed using the enhanced chemiluminescence substrate (ECL) (Millipore, USA). Signals were visualized and analyzed by the Bio-Rad Gel Imaging System on cool image workstation II (Viagene, USA).

### Apoptotic and cell cycle analysis

Cells were collected after been treated with DMSO, VP (10 μM) or VP combined with IM (2 μM) respectively for 48 h. Apoptosis was assessed by flow cytometry (FCM) using an Apoptosis Detection Kit (Becton-Dickinson) according to the manufacturer’s instruction. Moreover, cell morphology was examined by Wright’s staining and the results were observed with light microscope.

The cell cycle was analyzed by propidium iodide (PI) staining and quantified using FCM. The percentage of cells in different phases of the cell cycle was determined and quantitated by software (Becton, Dickinson, San Jose, CA, USA).

### MTT assay

For MTT assay, 2× 10^3^ of treated cells were seeded in per well of 96-well plates, then 50 μL MTT (2 mg/ml in PBS) (Sigma, USA) was added at indicated time, and incubated for 4 h at 37 °C. Then plates were centrifuged at 2000 rpm for 10 min. Supernatant was removed carefully. 150 μL of DMSO was added to each well. After shaking for 10 min the absorbance at 492 nm was measured by micro-plate reader (Eon, BioTeck, USA).

### Murine leukemogenesis model

2.0 × 10^7^ K562 cells were injected intravenously into NOD/SCID mice of 5–6 weeks old (*n* = 5, each group). From the second week, VP (50 mg/kg/mouse) was applied by intra-peritoneal (IP) and IM (100 mg/kg/mouse) was given by gavage. VP, IM and their combination were delivered every other day for four weeks while PBS was used as control. State, weight change and white blood cell counts of the mice were monitored weekly during administration of drugs. Furthermore, to detect the proportion of human-CD45 positive cells, peripheral blood cells were collected and white blood cells were separated from each group. The proportion of human-CD45 positive cells was detected by FCM.

### Statistical methods

Each experiment in this study was repeated for three times and values were summarized and represented as means ± standard deviations. The statistical analyses were performed by one-way ANOVA analysis to compare the mean of each group with that of the control group. All statistical analyses were performed using SPSS software. *p*-values < 0.05 was considered statistically significant.

## Results

### YAP is up-regulated in samples from CML patients and CML cell lines

**Fig. 1:**
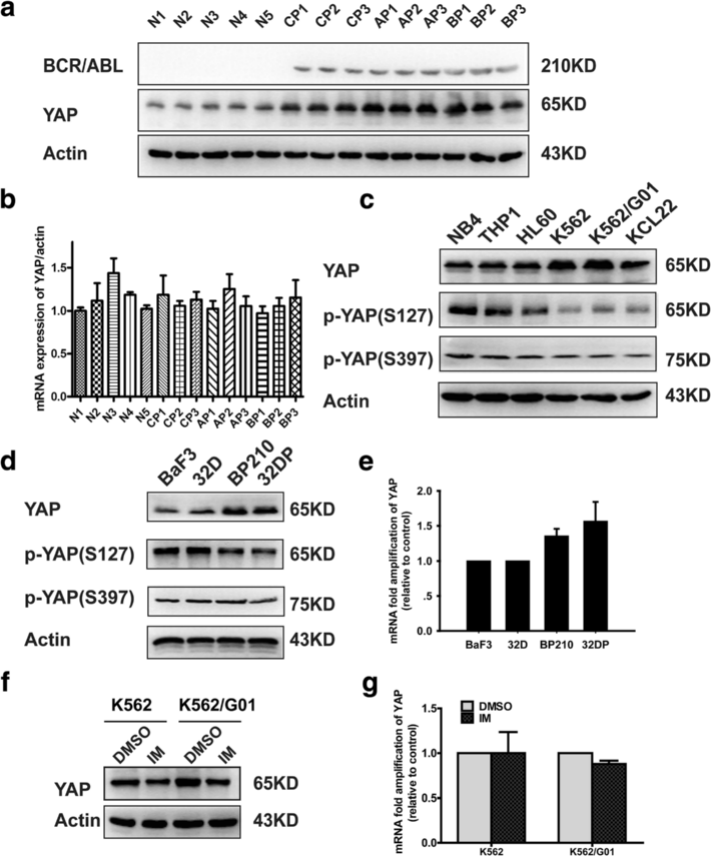
Expressions of YAP in BMMNCs from CML patients and different leukemic cell lines. **a** The protein expression of YAP was up-regulated in BMMNCs separated from CML patients in different phases (*n* = 9) compared with that from the healthy individuals (*n* = 5). **b** the mRNA level of YAP showed no significant difference between CML patients and the healthy individuals. **c** YAP was up-regulated in BCR/ABL positive cell lines K562, KCL22 and K562G01compared with BCR/ABL negative cell lines HL60, NB4 and THP. **d** The ectopic expression of BCR/ABL dramatically up-regulated YAP at protein level (**P < 0.01) but not mRNA level (**e**). Inhibition of BCR/ABL by IM down-regulated YAP at protein level (**f**) (**P < 0.01) but not mRNA level (**g**)

To determine the potential role of YAP in CML, we detected the expression of YAP in BMMNCs from healthy individuals (*n* = 5) and CML patients (*n* = 9) in different phases. Western blotting showed YAP was up-regulated in CML patients compared with the normal controls (Fig. 1a). RT-PCR showed no significant difference between each group (Fig. 1b). Next, we determined the expression of YAP in BCR/ABL^+^ leukemia cell lines (K562, KCL22 and K562/G01) and BCR/ABL^−^ cell lines (HL60, NB4 and THP1). Western blotting showed the protein level of YAP was much higher in BCR/ABL^+^ leukemia cell lines than that in BCR/ABL^−^ ones (Fig. 1c).

Previous studies have showed that c-Abl kinase activity can be activated in response to DNA damage [38]. Under this condition, activated c-Abl phosphorylates and stabilizes YAP in a kinase-dependent manner [39]. In CML, BCR-ABL is constitutively active. To confirm whether the up-regulation of YAP detected in BCR/ABL^+^ cells is associated with the tyrosine kinase activity of BCR/ABL, we compared the level of YAP in 32D, BaF3 and BCR/ABL transformed 32DP and BP210 cells. Furthermore, IM (2 μM) was used to inhibit the activity of BCR/ABL. The results indicated that the ectopic expression of BCR/ABL up-regulated protein level of YAP (Fig. 1d) but not mRNA level (Fig. 1e) and inhibition of BCR/ABL by IM reduced the expression of YAP at protein level (Fig. 1f) but not at transcription level (Fig. 1g). Here we found the expression of p-YAP(S127) increased first and then decreased after treated by IM (Additional file 1: Figure S1D). Overall, the results suggest that YAP might be involved in CML.

### Silencing of YAP inhibits the proliferation of CML cells

**Fig. 2:**
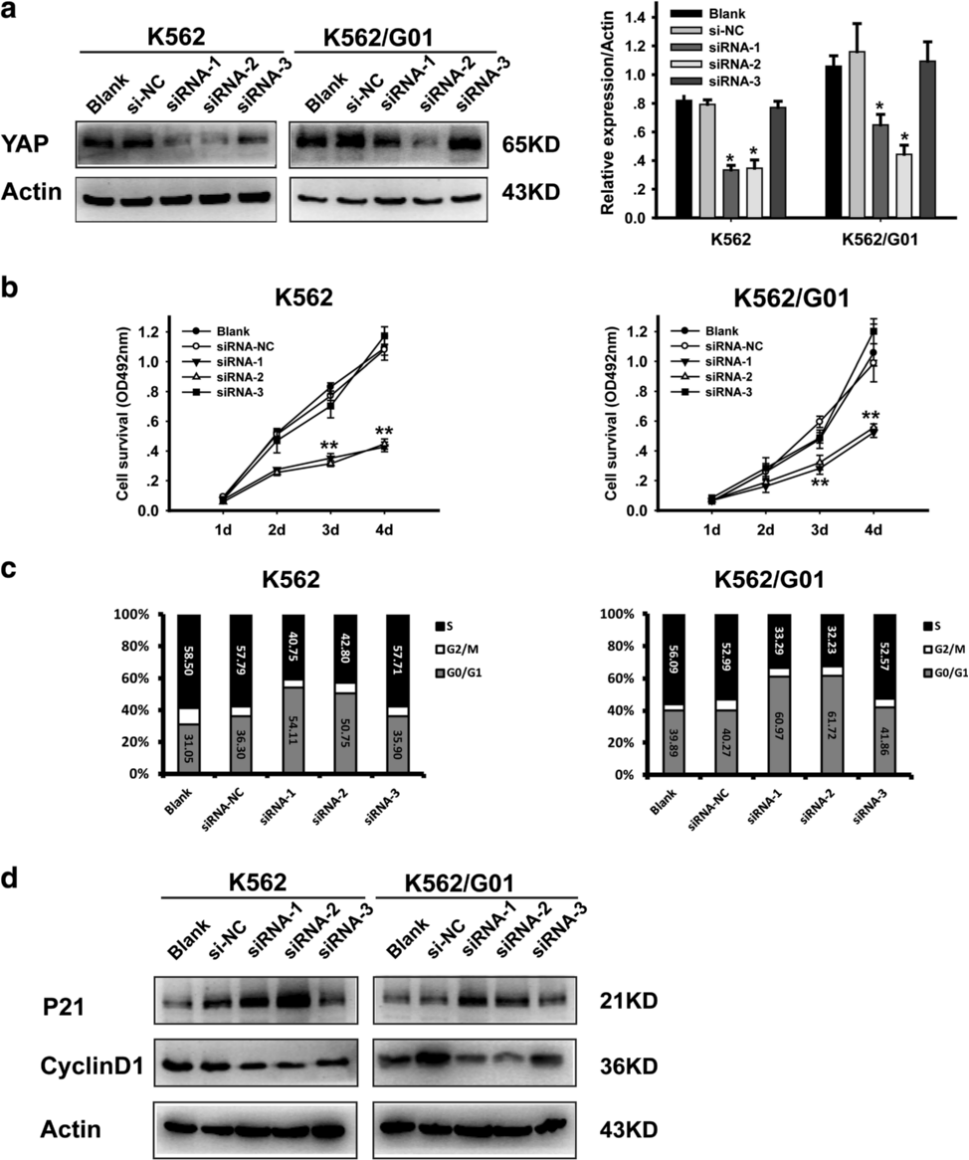
Silencing of YAP inhibits the proliferation of CML cells. **a** Knockdown efficiency of siRNA was validated by western blotting analysis. **b** Cell viability was determined by MTT, viability of siRNA transfected K562 and K562/G01 decreased in a time dependent manner (**p < 0.01). **c** Cell cycle was analyzed by flow cytometry at 48 h after transfection. **d** The expression of P21 and Cyclin D1 were analyzed by western blot

YAP-targeted therapy has obtained positive effects in various malignant cells [40]. Here we assessed the therapeutic potential of YAP in CML cell lines K562 and IM-resistant K562/G01. siRNA was used to knockdown YAP. The knockdown efficiency was examined by RT-PCR and western blotting (Fig. 2a). ctgf and cyr61 were detected to confirm the knockdown efficiency of siRNA (Additional file 1: Figure S1A, B). MTT showed that silencing of YAP significantly suppressed the proliferation of CML cells (Fig. 2b). FCM analysis demonstrated that knockdown of YAP induced blockade of cell cycle progression from G1 to S phase (Fig. 2c). Moreover, the expression of Cyclin D1 was decreased and the expression of p21 was increased in YAP silenced K562 and K562/G01cells (Fig. 2d).

### Silencing of YAP induces apoptosis of CML cells

**Fig. 3:**
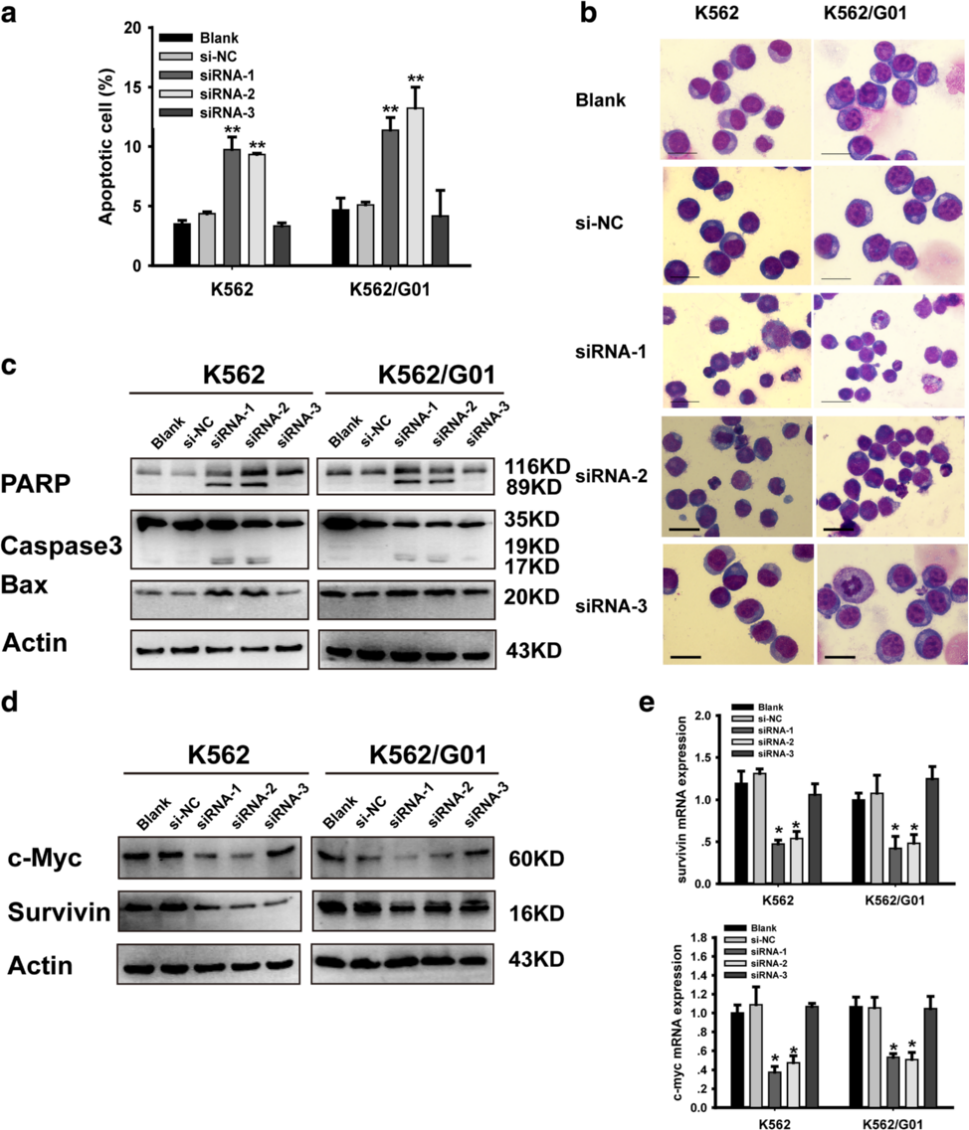
Silencing of YAP induces apoptosis of CML cells. **a** The percentage of apoptotic cells were examined by FCM. **b** Morphological features of the cell apoptosis induced by si-YAP (50 nM). **c** The expression of Bax, cleaved caspase-3 and PARP were analyzed by western blot. Knockdown of YAP by siRNA down-regulated c-Myc and survivin both at protein (**d**) and mRNA levels (**e**) (**P < 0.01)

Results of FCM showed that knockdown of YAP promotes apoptosis in CML cells (Fig. 3a). YAP silenced K562 and K562/G01 cells exhibited apoptosis characteristic morphological changes (Fig. 3b). Western blot analysis showed that the expression of proteins associated with apoptosis such as Bax, Caspase-3 and PARP cleavage products were increased in YAP silenced CML cells (Fig. 3c). RT-PCR and western blotting analysis were conducted to verify whether the expression of c-Myc and survivin, co-target genes of BCR-ABL and Hippo/YAP pathway, were changed by YAP silencing. The results illustrated that knockdown of YAP down-regulated c-Myc and survivin both at protein (Fig. 3d) and mRNA levels (Fig. 3e). These results indicated that after inducing cell cycle arrest, silencing of YAP triggered apoptosis of CML cells via regulating the expression of apoptosis-related proteins.

### YAP inhibitor enhances the effect of IM on CML cells

**Fig. 4:**
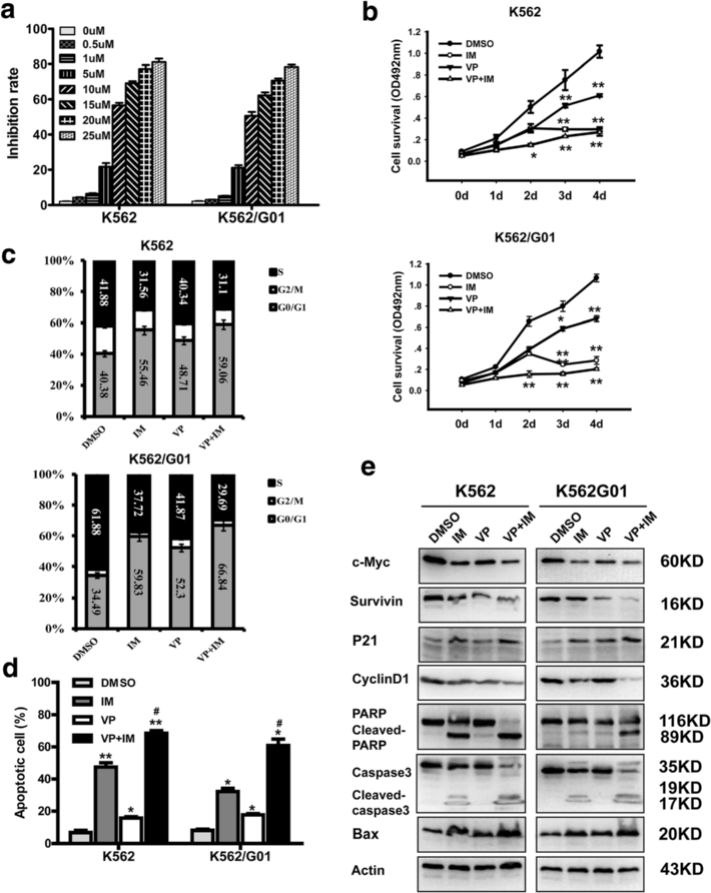
YAP inhibitor enhances the effect of IM on CML cells. **a** K562 and K562/G01 cells were treated with different dose of VP and the inhibitory rate was increased as a dose-dependent manner. **b** K562 and K562/G01 cells were treated with VP (10 μM) with or without 2 μM IM for 24 h, and cell viability was evaluated by MTT assay. **c** Cell cycle distribution was examined by FCM. **d** Apoptotic rate was analyzed by FCM. **e** After K562 and K562/G01cells were treated with VP, IM or their combination for 24 h, the expression of c-Myc, survivin, P21, Cyclin D1, Bax and the Cleaved caspase-3 and PARP were detected by western blot. *p < 0.05 and **p < 0.01 versus control group, #*p* < 0.05 versus IM alone

VP is a kind of inhibitor which can block the association of TEAD and YAP. Disruption of the TEAD-YAP complex suppresses the transcription of downstream genes [41]. It has been demonstrated that VP can sensitize cells to cytotoxics [42, 43]. To investigate the function of VP in CML cells, the concentration of VP used in our study was determined. K562 and K562/G01 were treated with VP at a series of concentrations (0.0 μM, 0.5 μM, 1 μM, 5 μM, 10 μM, 15 μM, 20 μM, 25 μM) for 48 h. The 50 % inhibitive concentration (IC50) was detected by MTT assay. The result showed that the IC50 value of K562 and K562/G01 was 10.281 ± 1.485 μM and 11.433 ± 1.675 μM, respectively (Fig. 4a). To explore whether the inhibition of YAP by VP can enhance the effect of IM on K562 and K562/G01, cells were treated with IM (2 μM), VP (10 μM) and IM combined with VP respectively. Compared with DMSO group, cells in IM and VP groups showed a decreased proliferation rate. The combination of VP and IM dramatically inhibited cell proliferation (Fig. 4b). Cell cycle analysis showed that VP promoted cell cycle arrest in G0/G1 phase induced by IM (Fig. 4c). VP remarkably facilitated IM induced apoptosis in CML cells (Fig. 4d and Additional file 1: Figure S1C). Meanwhile, to reveal the molecular mechanisms involved in YAP inhibition-mediated cell cycle arrest and apoptosis promotion, western blotting was conducted and the expression of associated proteins were detected. As shown in Fig. 4e, the combination of VP and IM induced down-regulation of c-Myc, survivin, Cyclin D1 and up-regulation of p21, Bax, Caspase-3 and PARP cleavage products compared to the treatment with IM or VP alone.

### YAP inhibitor potentiates the efficacy of IM on leukemogenesis in vivo

**Fig. 5:**
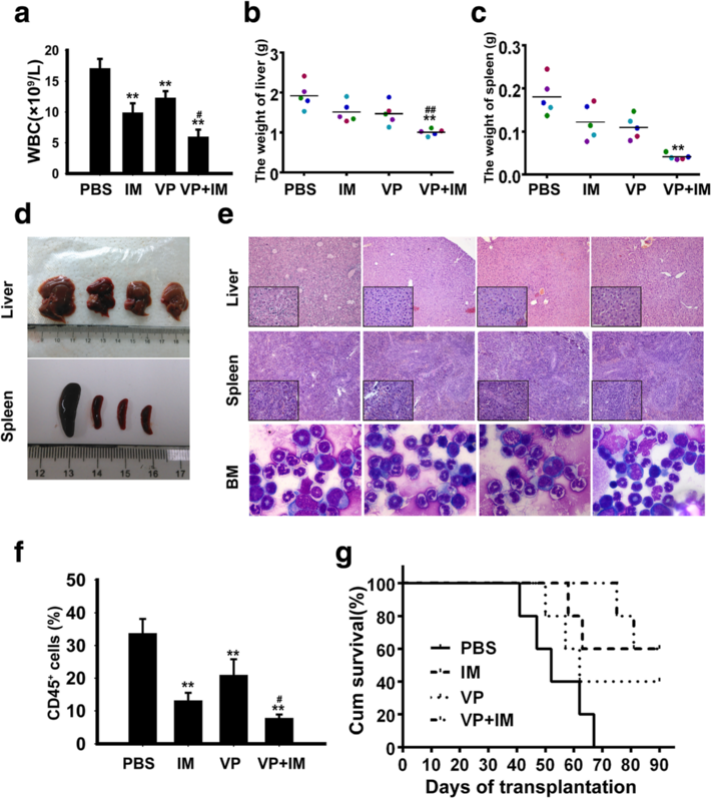
YAP inhibitor potentiates the efficacy of IM on leukemogenesis in vivo. **a** Total WBC count in PBS, VP, VP and IM group were determined. **b**, **c** Mean liver and spleen weight of mice in different group were quantified. **d** Representative images of livers and spleens from four groups. **e** Histologic sections of liver and spleen were stained with H&E and bone marrow cells from mice in each group were stained with Wright’s staining. **f** Percentage of Human CD45^+^ cells in murine bone marrow were detected by flow cytometry. **g** Survival curves were analyzed by Kaplan–Meier methods. *p < 0.05 and **p < 0.01 versus control group, #*p* < 0.05 and ##*p* < 0.01 versus IM alone

Whether inhibition of YAP could enhance the effect of IM on leukemogenesis induced by K562 cells was examined. First, the same numbers of K562 (2 × 10^7^) were injected into NOD-SCID mice intravenously. One week later, mice were treated with PBS, IM, VP and VP combined with IM respectively. The results showed that mice of PBS group displayed higher white blood cell counts compared with mice of IM, VP and the combination groups (Fig. 5a). Liver and spleen were excised and weighted. As shown in Fig. 5b, c and d, mice in PBS group have more severe splenomegaly and hepatomegaly compared with IM and VP groups. The combination of VP and IM partly alleviates these phenomenons.

Infiltration of leukemic cells in the liver and spleen was also examined by hematoxylin/eosin (HE) staining. Mice treated with IM, VP or their combination were observed with a reduced leukemic cell infiltration (Fig. 5e). Morphology of bone marrow also showed a decreased Myeloid : Erythroid ratio in mice treated with IM, VP and both of them (Fig. 5e). Peripheral blood cells were collected from each group and the proportion of human-CD45 positive cells which indicates the number of leukemic cells in murine bone marrow was detected by FCM. A significant reduction of CD45 positive cells was observed in the IM + VP group compared with IM and VP groups (Fig. 5f). Survival time of mice in combination group was significantly prolonged compared with the other groups (Fig. 5g). The mRNA expression level of YAP was detected, but no significant difference was found (Additional file 1: Figure S1E). The remaining samples were not enough for performing western blot, so we did not determin the protein level of YAP. These results further confirmed that inhibition of YAP by VP significantly increases the cytotoxicity of IM in vivo.

## Discussion

Our study revealed that YAP might be involved in the pathogenesis of CML and could be a potential target for the treatment of CML. We showed that YAP had a elevated level both in CML cell lines and BMMNCs from CML patients. Pharmacological or genetic inhibition of YAP efficaciously suppressed cell proliferation, induced cell cycle arrest in G1 phase and promoted apoptosis in CML cells. Moreover, the results derived from in vivo leukemogenesis assay also confirmed that inhibition of YAP enhanced the effects of IM on CML cells. Results of our study showed that the protein expression level of YAP was higher in BCR/ABL positive cell lines compared with BCR/ABL negative ones but there was no significant difference in the transcription level of YAP between them. Inhibition of BCR/ABL kinase activity by IM decreased protein level of YAP.

Previous study has demonstrated the activation of c-Abl induced by DNA damage antagonizes YAP oncogenic function via phosphorylating it at the Y357 residue [39, 44]. Different with c-Abl which is activated by DNA damage [45] and shuttles between the nucleus and cytoplasm [46], BCR/ABL mainly localizes in the cytoplasm and has constitutive activity [47]. As mentioned above, BCR/ABL activates PI3K-AKT and MEK-ERK pathways and both of them take part in the regulation of YAP. Activation of EGFR-PI3K-Akt signaling pathway up-regulates the expression of YAP, promotes its translocation into the nucleus to regulate the transcription of several downstream genes in diabetic mouse renal proximal tubule epithelial cells [48]. ERK1/2 inhibition decreases YAP protein level and down-regulates transcriptional activity of the Hippo pathway in non small cell lung cancer (NSCLC) cell lines [49]. These results suggest that BCR/ABL might influence the protein level of YAP through activating downstream pathways and influencing its stability, but the exact mechanism needs to be further investigated.

As a kinase of Hippo pathway, Lats2 has been demonstrated to prevent apoptosis induced by DNA damage through inhibition of c-Abl [50]. Although both of c-Abl and BCR/ABL have SH3 domain which could bind to Lats2, the function of Lats2 was not clear in CML. Former research has demonstrated that the expression of Lats2 was downregulated in K562 cells [51], which suggested that maybe in CML cells low level of Lats2 influenced its function.

It has been reported that YAP functions as an oncoprotein [26, 27, 52–54] by interacting with TEAD [55], forming a protein complex which is critical for the transcription of downstream genes such as c-Myc, and survivin [56, 57]. Porphyrin family members like VP, hematoporphyrin (HP) and protoporphyrin IX (PPIX) have been found as inhibitors of YAP through abrogating the interaction between YAP and TEAD [41]. Inhibition of YAP by VP synergistically inhibits esophageal cancer (EC) cells growth when in combination with 5-FU in vitro and in vivo [43]. But in CML, the function of VP is not clear. Here, we demonstrate that VP suppresses proliferation of CML cell and enhances the efficacy of IM in vitro and in vivo.

Overexpression of YAP/TAZ and survivin is commonly seen in malignancies [58]. Silencing of YAP enhances the efficacy of survivin inhibitor, indicating that simultaneous targeting these two molecules may achieve better therapeutic effect [59]. In CML, both of c-Myc and survivin are involved in the progression of CML [35, 60]. c-Myc is up-regulated by BCR/ABL [36, 61]. IM down-regulates c-Myc in CML cell lines and higher level of c-Myc at diagnosis correlates with worse response to IM [37]. Besides, targeting survivin sensitizes CML cells to IM and other cytotoxic drugs [62, 63]. In this study, we found that both genetic and pharmacological inhibition of YAP markedly reduced the expression of c-Myc and survivin. These results indicate that targeting YAP might influence the response of CML cells to IM via down-regulating the expression of c-Myc and survivin.

## Conclusions

In summary, our results demonstrated for the first time that YAP is involved in the pathogenesis of CML. YAP-targeted therapy may be a promising strategy with a better therapeutic effect in the presence of pharmacological inhibitors of BCR/ABL like IM.

## Additional file

Additional file 1: (A), (B) Knockdown of YAP by siRNA down-regulated ctgf and cyr61 at mRNA levels (***P* < 0.01). (C) Morphological changes of cell apoptosis induced by VP (10 μM, 24 h) with or without IM (2 μM). (D) IM decreased the protein level of YAP while the expression of p-YAP(S127) increased first and then decreased. (E) The mRNA level of YAP in bone cells collected from each group and the results showed no significant difference. (TIF 1855 kb)

## Abbreviations

BMMNCs: Bone marrow mononuclear cells
CLL: Chronic lymphoblastic leukemia
CML: Chronic myeloid leukemia
FCM: Flow cytometry
HE: Hematoxylin/eosin
IM: Imatinib
NSCLC: Non small cell lung cancer
PI: Propidium iodide
TKIs: Tyrosine kinase inhibitors
VP: Verteporfin
YAP: Yes-associated protein

## References

[1] Prieto F, Egozcue J, Forteza G, Marco F (1970). Identification of the Philadelphia (Ph-1) chromosome. Blood.

[2] Rowley JD (1973). Letter: a new consistent chromosomal abnormality in chronic myelogenous leukaemia identified by quinacrine fluorescence and Giemsa staining. Nature.

[3] Heisterkamp N, Stephenson JR, Groffen J, Hansen PF, de Klein A, Bartram CR, Grosveld G (1983). Localization of the c-ab1 oncogene adjacent to a translocation break point in chronic myelocytic leukaemia. Nature.

[4] Ben-Neriah Y, Daley GQ, Mes-Masson AM, Witte ON, Baltimore D (1986). The chronic myelogenous leukemia-specific P210 protein is the product of the bcr/abl hybrid gene. Science.

[5] Lugo TG, Pendergast AM, Muller AJ, Witte ON (1990). Tyrosine kinase activity and transformation potency of bcr-abl oncogene products. Science.

[6] Naughton R, Quiney C, Turner SD, Cotter TG (2009). Bcr-Abl-mediated redox regulation of the PI3K/AKT pathway. Leukemia.

[7] Hoover RR, Gerlach MJ, Koh EY, Daley GQ (2001). Cooperative and redundant effects of STAT5 and Ras signaling in BCR/ABL transformed hematopoietic cells. Oncogene.

[8] Baum KJ, Ren R (2008). Effect of Ras inhibition in hematopoiesis and BCR/ABL leukemogenesis. J Hematol Oncol.

[9] Sillaber C, Gesbert F, Frank DA, Sattler M, Griffin JD (2000). STAT5 activation contributes to growth and viability in Bcr/Abl-transformed cells. Blood.

[10] Mauro MJ, Druker BJ (2001). STI571: targeting BCR-ABL as therapy for CML. Oncologist.

[11] Weisberg E, Manley PW, Cowan-Jacob SW, Hochhaus A, Griffin JD (2007). Second generation inhibitors of BCR-ABL for the treatment of imatinib-resistant chronic myeloid leukaemia. Nat Rev Cancer.

[12] Tumaneng K, Russell RC, Guan KL (2012). Organ size control by Hippo and TOR pathways. Curr Biol.

[13] Piccolo S, Dupont S, Cordenonsi M (2014). The biology of YAP/TAZ: hippo signaling and beyond. Physiol Rev.

[14] Tremblay AM, Camargo FD (2012). Hippo signaling in mammalian stem cells. Semin Cell Dev Biol.

[15] Zhao B, Wei X, Li W, Udan RS, Yang Q, Kim J, Xie J, Ikenoue T, Yu J, Li L, Zheng P, Ye K, Chinnaiyan A, Halder G, Lai ZC, Guan KL (2007). Inactivation of YAP oncoprotein by the Hippo pathway is involved in cell contact inhibition and tissue growth control. Genes Dev.

[16] Yu FX, Guan KL (2013). The Hippo pathway: regulators and regulations. Genes Dev.

[17] Meng Z, Moroishi T, Guan KL (2016). Mechanisms of Hippo pathway regulation. Genes Dev.

[18] John MAS, Tao W, Fei X, Fukumoto R, Carcangiu ML, Brownstein DG, Parlow AF, McGrath J, Xu T (1999). Mice deficient of Lats1 develop soft-tissue sarcomas, ovarian tumours and pituitary dysfunction. Nat Genet.

[19] Tapon N, Harvey KF, Bell DW, Wahrer DC, Schiripo TA, Haber D, Hariharan IK (2002). salvador Promotes both cell cycle exit and apoptosis in Drosophila and is mutated in human cancer cell lines. Cell.

[20] Takahashi Y, Miyoshi Y, Takahata C, Irahara N, Taguchi T, Tamaki Y, Noguchi S (2005). Down-regulation of LATS1 and LATS2 mRNA expression by promoter hypermethylation and its association with biologically aggressive phenotype in human breast cancers. Clin Cancer Res.

[21] Zhang W, Gao Y, Li F, Tong X, Ren Y, Han X, Yao S, Long F, Yang Z, Fan H, Zhang L, Ji H (2015). YAP promotes malignant progression of Lkb1-deficient lung adenocarcinoma through downstream regulation of survivin. Cancer Res.

[22] Ma K, Xu Q, Wang S, Zhang W, Liu M, Liang S, Zhu H, Xu N. Nuclear accumulation of yes-associated protein (YAP) maintains the survival of doxorubicin-induced senescent cells by promoting survivin expression. Cancer Lett. 2016;375:84–91. 10.1016/j.canlet.2016.02.045 26944315

[23] Pobbati AV, Hong W (2013). Emerging roles of TEAD transcription factors and its coactivators in cancers. Cancer Biol Ther.

[24] Pei T, Li Y, Wang J, Wang H, Liang Y, Shi H, Sun B, Yin D, Sun J, Song R, Pan S, Sun Y, Jiang H, Zheng T, Liu L. YAP is a critical oncogene in human cholangiocarcinoma. Oncotarget. 2015;6:17206–17220. 10.18632/oncotarget.4043 PMC4627302 26015398

[25] Hall CA, Wang R, Miao J, Oliva E, Shen X, Wheeler T, Hilsenbeck SG, Orsulic S, Goode S (2010). Hippo pathway effector Yap is an ovarian cancer oncogene. Cancer Res.

[26] Lee KW, Lee SS, Kim SB, Sohn BH, Lee HS, Jang HJ, Park YY, Kopetz S, Kim SS, Oh SC, Lee JS (2015). Significant association of oncogene YAP1 with poor prognosis and cetuximab resistance in colorectal cancer patients. Clin Cancer Res.

[27] Xu MZ, Yao TJ, Lee NP, Ng IO, Chan YT, Zender L, Lowe SW, Poon RT, Luk JM (2009). Yes-associated protein is an independent prognostic marker in hepatocellular carcinoma. Cancer.

[28] Kang W, Tong JH, Chan AW, Lee TL, Lung RW, Leung PP, So KK, Wu K, Fan D, Yu J, Sung JJ, To KF (2011). Yes-associated protein 1 exhibits oncogenic property in gastric cancer and its nuclear accumulation associates with poor prognosis. Clin Cancer Res.

[29] Camargo FD, Gokhale S, Johnnidis JB, Fu D, Bell GW, Jaenisch R, Brummelkamp TR (2007). YAP1 increases organ size and expands undifferentiated progenitor cells. Curr Biol.

[30] Lian I, Kim J, Okazawa H, Zhao J, Zhao B, Yu J, Chinnaiyan A, Israel MA, Goldstein LS, Abujarour R, Ding S, Guan KL (2010). The role of YAP transcription coactivator in regulating stem cell self-renewal and differentiation. Genes Dev.

[31] Ramalho-Santos M, Yoon S, Matsuzaki Y, Mulligan RC, Melton DA (2002). “Stemness”: transcriptional profiling of embryonic and adult stem cells. Science.

[32] Yu Z, Yi S, Zhang Y, Li Z, Qiu L (2015). Expression of LATS mRNA in mantle cell lymphoma and its clinical significance. Zhonghua Yi Xue Za Zhi.

[33] Cottini F, Hideshima T, Xu C, Sattler M, Dori M, Agnelli L, ten Hacken E, Bertilaccio MT, Antonini E, Neri A, Ponzoni M, Marcatti M, Richardson PG, Carrasco R, Kimmelman AC, Wong KK, Caligaris-Cappio F, Blandino G, Kuehl WM, Anderson KC, Tonon G (2014). Rescue of Hippo coactivator YAP1 triggers DNA damage-induced apoptosis in hematological cancers. Nat Med.

[34] Badran A, Yoshida A, Wano Y, Imamura S, Kawai Y, Tsutani H, Inuzuka M, Ueda T (2003). Expression of the antiapoptotic gene survivin in chronic myeloid leukemia. Anticancer Res.

[35] Hernandez-Boluda JC, Bellosillo B, Vela MC, Colomer D, Alvarez-Larran A, Cervantes F (2005). Survivin expression in the progression of chronic myeloid leukemia: a sequential study in 16 patients. Leuk Lymphoma.

[36] Gomez-Casares MT, Vaque JP, Lemes A, Molero T, Delgado MD, Leon J (2004). C-myc expression in cell lines derived from chronic myeloid leukemia. Haematologica.

[37] Albajar M, Gomez-Casares MT, Llorca J, Mauleon I, Vaque JP, Acosta JC, Bermudez A, Donato N, Delgado MD, Leon J (2011). MYC in chronic myeloid leukemia: induction of aberrant DNA synthesis and association with poor response to imatinib. Mol Cancer Res.

[38] Shaul Y, Ben-Yehoyada M (2005). Role of c-Abl in the DNA damage stress response. Cell Res.

[39] Levy D, Adamovich Y, Reuven N, Shaul Y (2008). Yap1 phosphorylation by c-Abl is a critical step in selective activation of proapoptotic genes in response to DNA damage. Mol Cell.

[40] Felley-Bosco E, Stahel R (2014). Hippo/YAP pathway for targeted therapy. Transl Lung Cancer Res.

[41] Liu-Chittenden Y, Huang B, Shim JS, Chen Q, Lee SJ, Anders RA, Liu JO, Pan D (2012). Genetic and pharmacological disruption of the TEAD-YAP complex suppresses the oncogenic activity of YAP. Genes Dev.

[42] Ciamporcero E, Shen H, Ramakrishnan S, Yu Ku S, Chintala S, Shen L, Adelaiye R, Miles KM, Ullio C, Pizzimenti S, Daga M, Azabdaftari G, Attwood K, Johnson C, Zhang J, Barrera G, Pili R. YAP activation protects urothelial cell carcinoma from treatment-induced DNA damage. Oncogene. 2015;35:1541–1553. 10.1038/onc.2015.219 PMC4695331 26119935

[43] Song S, Honjo S, Jin J, Chang SS, Scott AW, Chen Q, Kalhor N, Correa AM, Hofstetter WL, Albarracin CT, Wu TT, Johnson RL, Hung MC, Ajani JA (2015). The hippo coactivator YAP1 mediates EGFR overexpression and confers chemoresistance in esophageal cancer. Clin Cancer Res.

[44] Keshet R, Adler J, Ricardo Lax I, Shanzer M, Porat Z, Reuven N, Shaul Y (2015). c-Abl antagonizes the YAP oncogenic function. Cell Death Differ.

[45] Baskaran R, Wood LD, Whitaker LL, Canman CE, Morgan SE, Xu Y, Barlow C, Baltimore D, Wynshaw-Boris A, Kastan MB, Wang JY (1997). Ataxia telangiectasia mutant protein activates c-Abl tyrosine kinase in response to ionizing radiation. Nature.

[46] Taagepera S, McDonald D, Loeb JE, Whitaker LL, McElroy AK, Wang JY, Hope TJ (1998). Nuclear-cytoplasmic shuttling of C-ABL tyrosine kinase. Proc Natl Acad Sci U S A.

[47] McWhirter JR, Wang JY (1993). An actin-binding function contributes to transformation by the Bcr-Abl oncoprotein of Philadelphia chromosome-positive human leukemias. EMBO J.

[48] Chen J, Harris RC. Interaction of the EGF Receptor and the Hippo Pathway in the Diabetic Kidney. J Am Soc Nephrol. 2015;27:1689–1700. 10.1681/ASN.2015040415 PMC4884112 26453611

[49] You B, Yang YL, Xu Z, Dai Y, Liu S, Mao JH, Tetsu O, Li H, Jablons DM, You L (2015). Inhibition of ERK1/2 down-regulates the Hippo/YAP signaling pathway in human NSCLC cells. Oncotarget.

[50] Reuven N, Adler J, Meltser V, Shaul Y (2013). The Hippo pathway kinase Lats2 prevents DNA damage-induced apoptosis through inhibition of the tyrosine kinase c-Abl. Cell Death Differ.

[51] Kawahara M, Hori T, Chonabayashi K, Oka T, Sudol M, Uchiyama T (2008). Kpm/Lats2 is linked to chemosensitivity of leukemic cells through the stabilization of p73. Blood.

[52] Fernandez LA, Northcott PA, Dalton J, Fraga C, Ellison D, Angers S, Taylor MD, Kenney AM (2009). YAP1 is amplified and up-regulated in hedgehog-associated medulloblastomas and mediates Sonic hedgehog-driven neural precursor proliferation. Genes Dev.

[53] Li SY, Hu JA, Wang HM (2013). Expression of Yes-associated protein 1 gene and protein in oral squamous cell carcinoma. Chin Med J.

[54] Liu JY, Li YH, Lin HX, Liao YJ, Mai SJ, Liu ZW, Zhang ZL, Jiang LJ, Zhang JX, Kung HF, Zeng YX, Zhou FJ, Xie D (2013). Overexpression of YAP 1 contributes to progressive features and poor prognosis of human urothelial carcinoma of the bladder. BMC Cancer.

[55] Zhao B, Ye X, Yu J, Li L, Li W, Li S, Yu J, Lin JD, Wang CY, Chinnaiyan AM, Lai ZC, Guan KL (2008). TEAD mediates YAP-dependent gene induction and growth control. Genes Dev.

[56] Xiao W, Wang J, Ou C, Zhang Y, Ma L, Weng W, Pan Q, Sun F (2013). Mutual interaction between YAP and c-Myc is critical for carcinogenesis in liver cancer. Biochem Biophys Res Commun.

[57] Liang Y, Xu RZ, Zhang L, Zhao XY (2009). Berbamine, a novel nuclear factor kappaB inhibitor, inhibits growth and induces apoptosis in human myeloma cells. Acta Pharmacol Sin.

[58] Steinhardt AA, Gayyed MF, Klein AP, Dong J, Maitra A, Pan D, Montgomery EA, Anders RA (2008). Expression of Yes-associated protein in common solid tumors. Hum Pathol.

[59] Huang JM, Nagatomo I, Suzuki E, Mizuno T, Kumagai T, Berezov A, Zhang H, Karlan B, Greene MI, Wang Q (2013). YAP modifies cancer cell sensitivity to EGFR and survivin inhibitors and is negatively regulated by the non-receptor type protein tyrosine phosphatase 14. Oncogene.

[60] Karasawa M, Okamoto K, Maehara T, Tsukamoto N, Morita K, Naruse T, Omine M (1996). Detection of c-myc oncogene amplification in a CML blastic phase patient with double minute chromosomes. Leuk Res.

[61] Xie S, Lin H, Sun T, Arlinghaus RB (2002). Jak2 is involved in c-Myc induction by Bcr-Abl. Oncogene.

[62] Carter BZ, Mak DH, Schober WD, Cabreira-Hansen M, Beran M, McQueen T, Chen W, Andreeff M (2006). Regulation of survivin expression through Bcr-Abl/MAPK cascade: targeting survivin overcomes imatinib resistance and increases imatinib sensitivity in imatinib-responsive CML cells. Blood.

[63] Stella S, Tirro E, Conte E, Stagno F, Di Raimondo F, Manzella L, Vigneri P (2013). Suppression of survivin induced by a BCR-ABL/JAK2/STAT3 pathway sensitizes imatinib-resistant CML cells to different cytotoxic drugs. Mol Cancer Ther.

