# Supplementary figures and images for: Inhibition of YAP suppresses CML cell proliferation and enhances efficacy of imatinib in vitro and in vivo
Source: J Exp Clin Cancer Res. 2016 Sep 6;35(1):134. doi: 10.1186/s13046-016-0414-z (PMC5012077; doi:10.1186/s13046-016-0414-z)

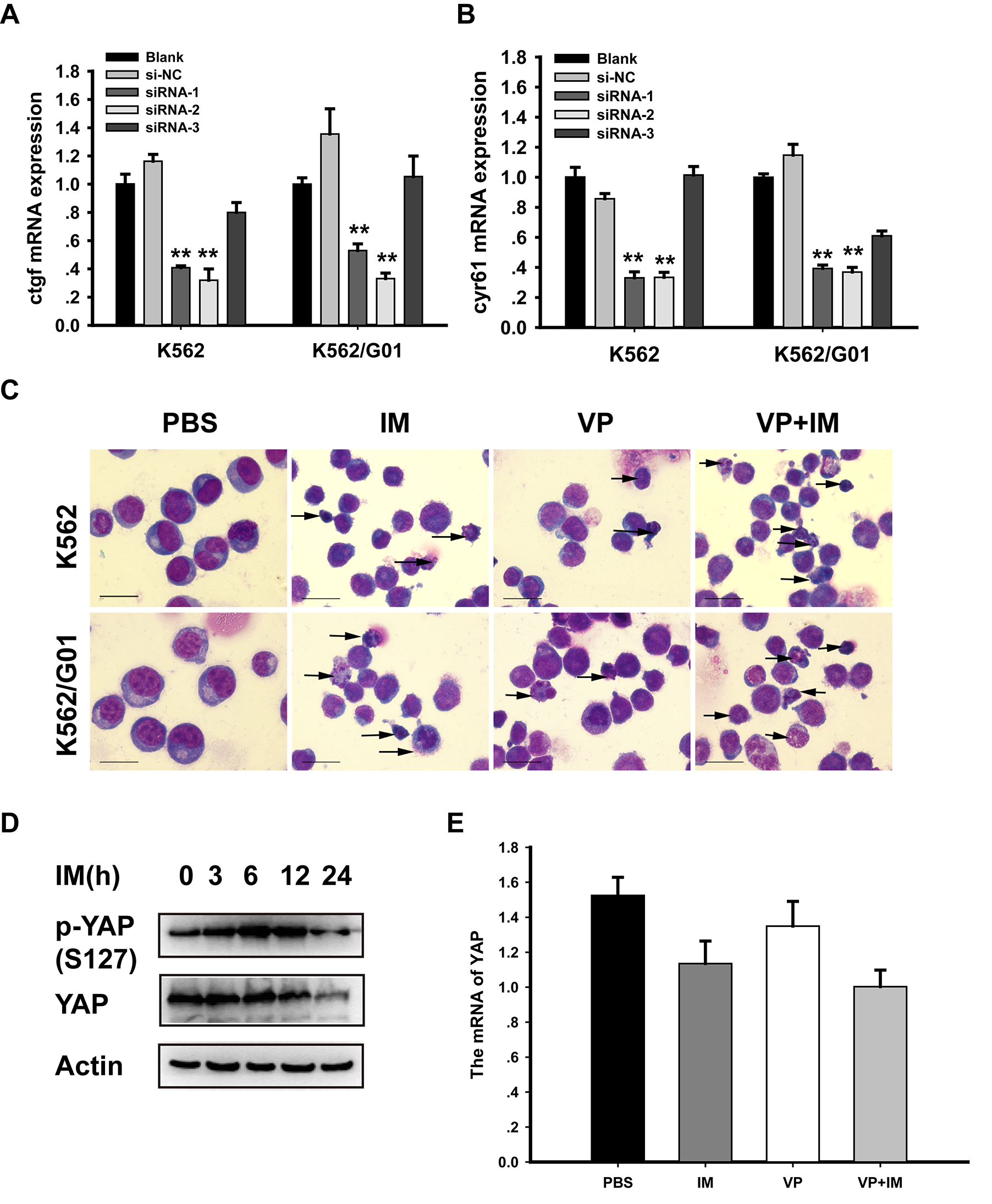

Supplement: Additional file 1: — (A), (B) Knockdown of YAP by siRNA down-regulated ctgf and cyr61 at mRNA levels (**P < 0.01). (C) Morphological changes of cell apoptosis induced by VP (10 μM, 24 h) with or without IM (2 μM). (D) IM decreased the protein level of YAP while the expression of p-YAP(S127) increased first and then decreased. (E) The mRNA level of YAP in bone cells collected from each group and the results showed no significant difference. (TIF 1855 kb) [file 13046_2016_414_MOESM1_ESM.tif]
